# Formulation and Characterization of Teicoplanin Niosomal Gel for Healing Chronic Wounds Infected with Methicillin-Resistant *Staphylococcus aureus* (MRSA)

**DOI:** 10.3390/gels11040230

**Published:** 2025-03-22

**Authors:** Jaber Hemmati, Iraj Sedighi, Mehdi Azizi, Zahra Chegini, Raha Zare Shahraki, Mohsen Chiani, Mohammad Reza Arabestani

**Affiliations:** 1 Department of Microbiology, School of Medicine, Hamadan University of Medical Sciences, Hamadan P.O. Box 6517838678, Iran; jaberhemmati@gmail.com (J.H.); parisa.chegini72@gmail.com (Z.C.); raha.zr.sh@gmail.com (R.Z.S.); 2 Student Research Committee, Hamadan University of Medical Sciences, Hamadan P.O. Box 4171-65175, Iran; 3 Department of Pediatrics, Faculty of Medicine, Hamadan University of Medical Sciences, Hamadan P.O. Box 4171-65175, Iran; doctorsedighi@yahoo.com; 4 Department of Tissue Engineering and Biomaterials, School of Advanced Medical Sciences and Technologies, Hamadan University of Medical Sciences, Hamadan P.O. Box 6517838736, Iran; mehdi.azizi6815@gmail.com; 5 Department of NanoBiotechnology, Pasteur Institute of Iran, Tehran P.O. Box 13169-43551, Iran; 6 Nutrition Health Research Center, Institute of Health Sciences and Technologies, Hamadan University of Medical Sciences, Hamadan P.O. Box 4171-65175, Iran

**Keywords:** niosomal gel, teicoplanin, biofilm, HPMC polymer, methicillin-resistant *Staphylococcus aureus* (MRSA), chronic wound

## Abstract

Methicillin-resistant *Staphylococcus aureus* (MRSA) is recognized as a significant pathogen playing a crucial role in causing bacterial infections of skin and soft tissues due to its high capacity for biofilm formation. Niosome-based gel systems offer significant potential for enhancing transdermal drug delivery and increasing the effectiveness of loaded drugs. The current research investigates the feasibility of niosomal gel for formulating the topical administration of teicoplanin (TEC). The thin film hydration method was used for niosome formulation was composed of nonionic surfactant, cholesterol, and mPEG 2000. TEC niosomal gel was prepared with adding hydroxypropyl methylcellulose (HPMC) and Poloxamer 407 polymers to the system. The physiochemical characteristics of prepared niosomal gel formulation, such as particle morphology, size, zeta surface charge, homogeneity, encapsulation efficiency, and in vitro drug release, were evaluated. Also, the in vitro antibacterial potential of the prepared system was analyzed. Further, we examined the in vivo antibacterial activity of the synthesized niosomal gel on infected wounds in Wister rats. We found that the TEC niosomal gel had antibacterial and anti-biofilm capabilities against MRSA isolates, and could be an effective wound material for preventing therapeutic problems related to this superbug.

## 1. Introduction

Full-thickness infected skin wounds necessitate the application of wound dressings to enhance healing and manage post-burn infections while minimizing scar formation [1,2,3]. Healing a deeply infected wound is a complex process that requires a supportive environment to facilitate tissue repair. Deep infections can significantly impede the healing process by promoting inflammation and tissue damage. Modern wound coatings are designed to create a moist environment that supports healing by allowing for gas exchange and protecting the wound from contaminants [4,5]. The development of coatings with optimal gas permeability and occlusivity is essential for managing deep infected wounds effectively, and could promote healing through enhanced cell migration and tissue regeneration [6,7]. Currently, transdermal drug delivery systems based on nanoparticles may offer a novel and highly effective strategy for addressing the therapeutic challenges associated with bacterial wound infections [8]. Also, different types of wound dressings have been developed using both synthetic and natural biomaterials, with some already in use in clinical settings [9,10,11].

The complete reconstruction of skin with full functionality remains a significant challenge, particularly in cases involving wounds infected with multi-drug-resistant (MDR) bacteria, which continues to be a major concern in health-care settings [9]. Wound infection represents a significant barrier to the natural healing process and can lead to severe health complications, such as bacteremia and septicemia, which may be life-threatening [12]. The presence of an invasive pathogen disrupts blood supply, prolongs the inflammatory phase, and ultimately delays normal healing. While the use of antibiotics remains the most effective approach for treating wounds, the emergence of MDR pathogens due to antibiotic misuse has raised global concerns. Consequently, recent studies have focused on developing innovative treatment methods for MDR-infected wounds as alternatives to traditional antibiotic therapies [13].

Methicillin-resistant *Staphylococcus aureus* (MRSA) is acknowledged as a critical pathogen that contributes to bacterial skin and soft tissue infections in individuals. This bacterium is among the leading pathogens that colonize wounds, resulting in chronic and potentially life-threatening infections, particularly in individuals with weakened immune systems [14,15]. Moreover, the swift emergence of antibiotic resistance in MRSA strains is complicating the management of wound infections linked to this pathogen [16]. MRSA exhibits a greater capacity for biofilm formation, significantly contributing to the development of emerging drug resistance. A biofilm is characterized as a community of living bacteria encased within an extracellular polymeric matrix (EPS), which enhances bacterial survival in challenging conditions, including exposure to elevated concentrations of antimicrobial agents [17]. This structure serves as a primary mechanism by which bacteria develop resistance to antimicrobial agents. The restricted penetration of drugs into the biofilm matrix enables the bacteria contained within to endure high concentrations of antibiotics, leading to chronic infections for which effective treatment options are frequently lacking [18]. Moreover, the bacterial biofilm that forms in chronic wounds impedes the distribution of systemic medications to the damaged skin. This situation emphasizes the critical necessity for effective topical drug delivery in the management of chronic wound infections.

Niosome-based gel systems have the potential to improve the transdermal delivery of drugs and enhance the efficacy of antibiotics in the treatment of bacterial wound infections. Also, niosomal gel has the capacity to improve the permeation behavior of encapsulated drugs while providing controlled therapeutic activity and prolonged residence time. Additionally, the studies approved that niosomal gels with more physical stability, appropriate rheological characterization, and excellent antimicrobial effectiveness compared with conventional topical approaches could be a novel option against *S. aureus* infections. Consequently, this system can serve as a potent transdermal nano-carrier and may offer a novel approach to the treatment of bacterial skin infections [19,20].

Teicoplanin (TEC) is selected as an antibacterial candidate to treat infections of the skin caused by MRS strains. Clinically, TEC exhibits a wide range of activity and is recommended as a preferred treatment option for severe MRSA infections, especially in patients at high risk of morbidity and mortality from wound infections. Research indicates that TEC possesses advantageous pharmacokinetic properties, including extensive tissue penetration, which are essential for the effective management of both skin and underlying tissues infections [21,22]. Recent clinical assessments indicate that increased dosing regimens may improve therapeutic outcomes, highlighting the significance of treatment strategies tailored to individual susceptibility profiles. Therefore, TEC could be considered a significant antimicrobial agent against MRSA infections, underscoring the need for ongoing research to optimize its application and effectively address resistance. Nonetheless, the emergence of resistance, adverse side effects such as potential nephrotoxicity and cutaneous reactions, along with the variable pharmacokinetics present challenges to the treatment efficacy of TEC [22].

The present research is based on the hypothesis that encapsulation of TEC in a niosomal gel system will enhance the amount and retention time of the drug within the skin; which would enhance the therapeutic efficacy of the drug through sustaining drug release. Also, we aimed to propose a novel healing agent against MRSA wound infections by in vitro and in vivo antimicrobial analyses. Therefore, the current research investigates the feasibility of niosomal gel for formulating the topical administration of TEC. A TEC niosomal gel was prepared by adding hydrophilic polymer to the system to improve the stability and increase the viscosity. Further, we examined the in vivo antibacterial activity of the synthesized TEC niosome gel in an animal wound model.

## 2. Results and Discussion

### 2.1. Physicochemical Characterization of Formulated Nanoparticle

#### 2.1.1. Morphology, Hydrodynamic Size, Surface Zeta Potential

**Figure 1:**
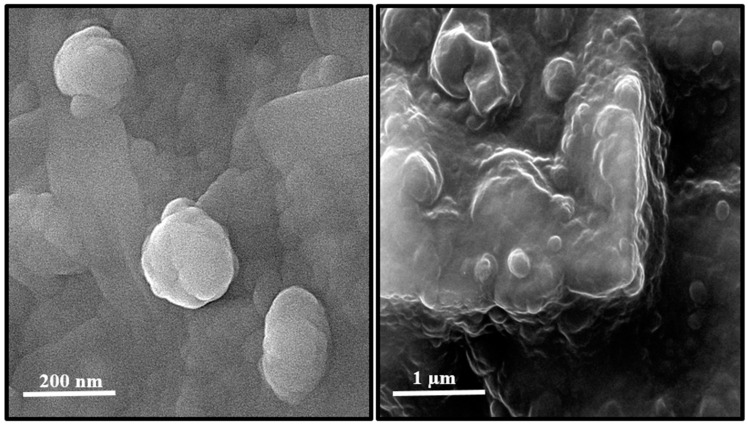
Structure of TEC niosomes (**left**) and TEC niosomal gel (**right**) formulations taken with FE-SEM.

Using field emission scanning electron microscope (FE-SEM), the morphology of TEC niosome and TEC niosomal gel were investigated (Figure 1). The FE-SEM micrograph showed the spherical structure TEC niosomes with an average size of 200 nm, which properly dispersed into the gel system.

**Figure 2:**
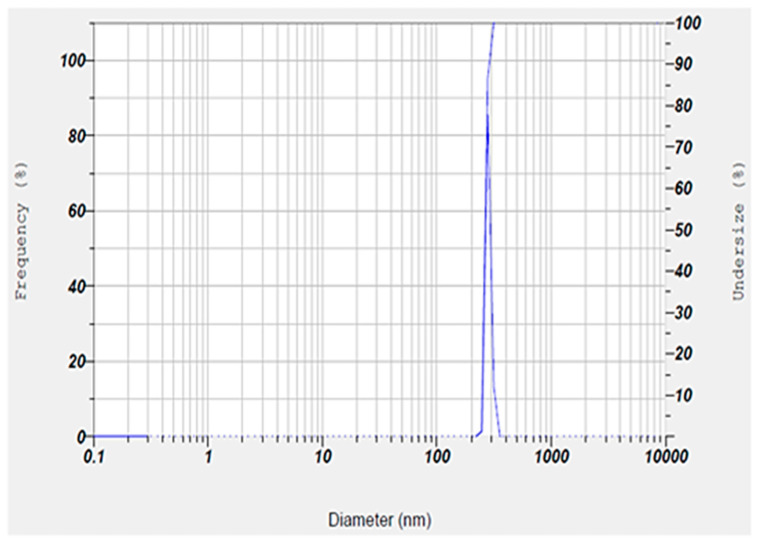
Size and homogeneity of niosomal gel obtained by DLS technique. The niosomal gel was monodispersed with about 300 nm particle size.

**Figure 3:**
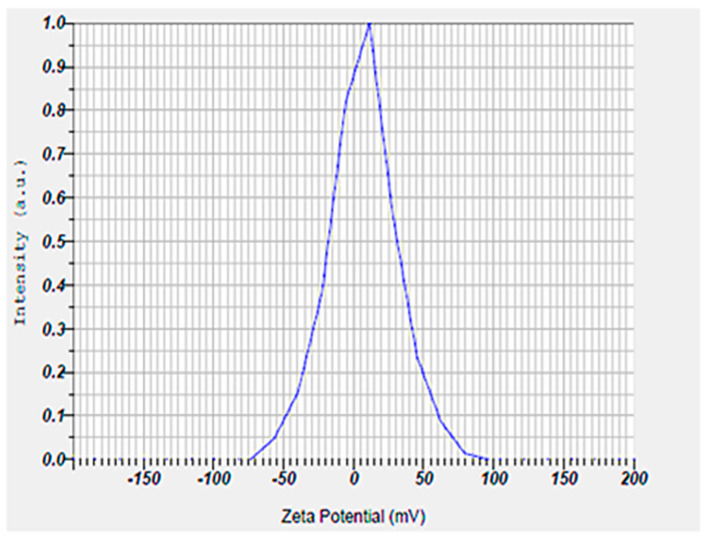
Surface zeta potential result of niosomal gel obtained by ELS technique. The niosomal gel showed a zeta potential around +7 mV.

Also, the hydrodynamic size, polydispersity index (PDI), and surface zeta potential of the niosomal gel formulation were measured to be 302.6 nm, 0.291, and +6.7 mV, respectively (Figure 2 and Figure 3).

The size of the nanoparticles measured by DLS was larger than that obtained from SEM analysis. This discrepancy is likely due to the fact that DLS measures the hydrodynamic diameter, while SEM provides the diameter of dried particles [23,24,25]. Also, our synthesized niosomes had an acceptable hydrodynamic size, which can improve the therapeutic outcomes by allowing for optimal interaction with microbial membranes. Additionally, niosomes in this size range tend to have a longer retention time at the application site, which could improve the therapeutic outcomes by maintaining a sustained release of encapsulated drugs. In addition, an appropriate hydrodynamic size offers several advantages for our niosomal formulation in wound healing by enhancing the delivery of therapeutic agents at the infected sites [26]. A possible reason for positive zeta potential is the surface modification of particles with mPEG2000. Furthermore, the measured PDI value of the synthesized niosomal gel was found to be a satisfactory homogeneous dispersion [27,28].

#### 2.1.2. Release Profile and Entrapment Efficiency (EE%)

In this study, the dynamic dialysis assay was employed to investigate the release kinetics of the niosomal gel formulation. For this purpose, release analysis data were analyzed using mathematical methods based on the kinetic models’ equations, including Korsmeyer–Peppas, the Weibull and Hyperbolic Tangent Function, and zero- to fifth-order polynomials [28,29,30].

**Figure 4:**
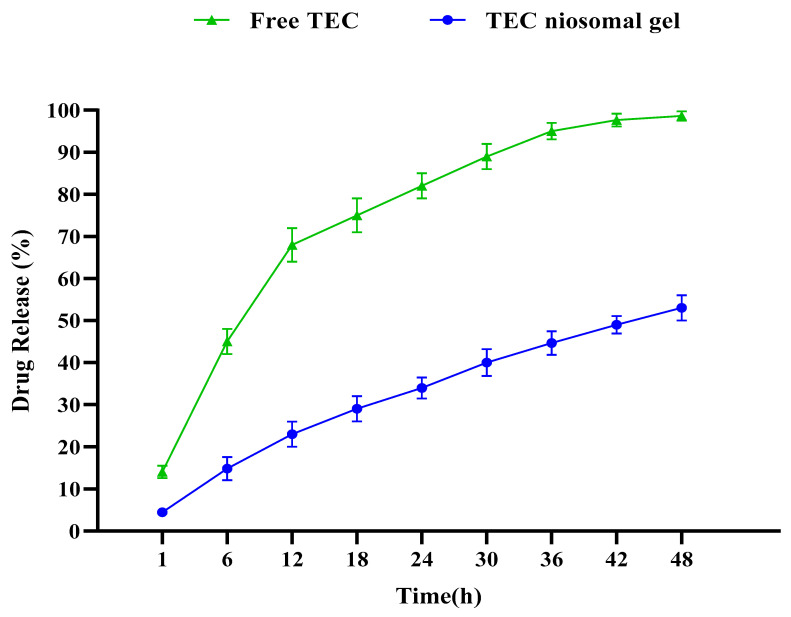
Comparative release profiles of both free TEC and TEC niosomal gel formulation. A suatained release profile was obtained for TEC niosomal gel.

Our statistical analysis revealed that the first-order kinetic model was the most appropriate for describing drug release kinetics from both free and niosomal gel formulations, and the TEC niosomal gel exhibited a greater sustained release range in comparison to the free TEC formulation (Figure 4). Our findings showed a burst drug release from the free TEC formulation during the first 12 h, with approximately 70%. After 12 h, the drug release phase had a slower range. Also, the drug release rates from the niosomal gel formulation were around 25%, 35%, and 55% during 12, 24, and 48 h, respectively.

The rate of TEC release from a niosomal gel is a considerable index influencing pharmaceutical properties. Additionally, the in vitro drug release analysis was performed to provide insights into the in vivo effectiveness of the synthesized niosomal formulation. By comparing TEC release ranges from both free TEC and TEC niosomal gel formulations, it was found that niosomal gel has a greater sustained release profile, which could hinder the burst drug release. Also, our formulation released the encapsulated drug for a longer period of time in comparison with other studies [31,32], which could be related to dispersing the synthesized niosome into a gel system. Also, our result was in line with another study in which the positive effect of hydroxypropyl methylcellulose (HPMC) and poloxamer 704 polymers on the release profile of drug delivery systems was approved [33].

Our formulation exhibited that the amount of TEC loaded into the niosomal gel was calculated to be 74.6%, representing a satisfactory EE rate of the synthesized niosomal formulation. Incorporation into the niosomal gel is known to increase the pharmaceutical ability of the encapsulated drug, considered a prominent index in the development of drug delivery systems [28]. Also, our formulation had a significant EE%, which could be attributed to the presence of long-chain non-ionic surfactant (span 60) and cholesterol in the niosomal formulation. Therefore, the proper selection of surfactant and the molar ratio of niosomal components are critical parameters that must be carefully considered to develop an optimized formulation.

#### 2.1.3. Stability Study

**Figure 5:**
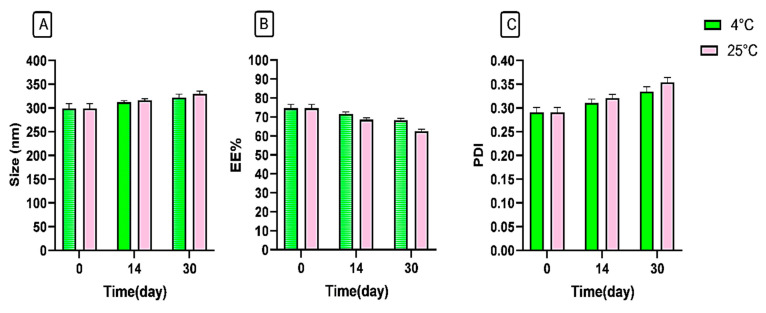
Stability analysis of niosomal gel formulation according to (**A**) size (nm), (**B**) EE%, and (**C**) PDI at 4 °C and 25 °C.

The physical stability of the synthesized niosomal gel was assessed over a 30-day period under two storage conditions by observing changes in hydrodynamic size, EE%, and PDI. Our findings showed that the refrigerated condition yielded superior stability, with minimal changes in the examined parameters compared to the room temperature storage condition (Figure 5). Notably, our formulation demonstrated greater stability than other formulations [34,35], which may be attributed to applying HPMC and Poloxamer 407 polymers into the system. Additionally, the synthesis methods, type of surfactant, molar ratio of components, and surface modification could play significant roles in enhancing the stability of nanoparticles [36,37,38].

### 2.2. Antimicrobial Analysis

#### 2.2.1. Determination of Minimum Inhibition/Bactericidal Concentration (MIC/MBC)

**Figure 6:**
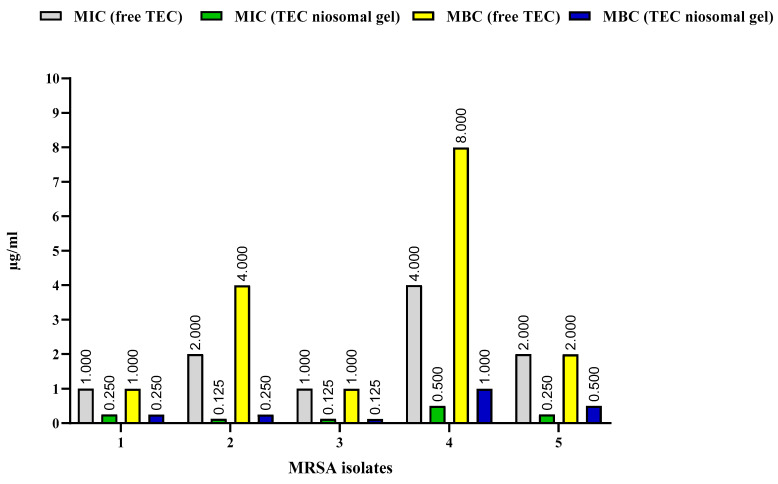
Comparison of MICs and MBCs of free TEC and TEC niosomal gel against 5 MRSA isolates.

The MIC and MBC values of the TEC niosomal gel against five MRSA strains were analyzed and were compared with the free TEC formulation (Figure 6). Our antimicrobial analysis showed that the preparation of the niosomal gel formulation reduced the MICs of the drug TEC against all MRSA isolates by 4–16-fold. In addition, the TEC niosomal gel had greater bactericidal ability in comparison to the free TEC formulation, which decreased the MBCs of the drug at lower concentrations. Notably, the antimicrobial analysis of blank niosomal gel was investigated, and it had no inhibitory or bactericidal effects against all tested strains.

By enhancing the effective dose of the encapsulated drug, niosomal encapsulation could be developed as a viable strategy for drug delivery targeting MRSA infections. Encapsulating antibiotics within niosomal nano-carriers offers advantages in improving their antibacterial potential with desirable outcomes [18,39]. In this regard, a study showed that the antimicrobial activity (MICs and MBCs) of drug-loaded niosome nanoparticles was 2–4-fold greater than that of the free drug against *S. aureus* and MSRA strains [40]. In another study, a synthesized niosomal formulation efficiently improved the MIC of fluoroquinolones at least 4-fold against *S. aureus* isolates with significant antibacterial abilities over conventional antibiotics [32]. Also, the scientists prepared ciprofloxacin-loaded niosome nanoparticles, which the optimized drug delivery system had an 8–32-fold greater activity against *Staphylococcus* spp. strains than the free formulation [41]. Additionally, the results of the study showed that niosomal gel containing azithromycin had good antibacterial activity against *S. aureus* [19]. Furthermore, the findings of another study showed that niosomal gel containing moxifloxacin could markedly increase the efficacy against *S. aureus* by reducing 2-fold the MIC of the free drug [42]. In line with these studies, the anti-MRSA effects of TEC niosomal gel were evalulated, and it was demonstrated that the niosomal drug delivery system holds promise as an alternative strategy, with a 4–16-fold reduction in both MIC and MBC values compared to the free formulation. The niosomal vesicular system is designed to deliver encapsulated antibiotics directly into bacterial cells by exploiting the fluidity of its bilayer properties [43]. This strategy seeks to reduce dose-dependent side effects while improving the effectiveness of antibiotic therapy. In Gram-positive bacteria, niosomes facilitate intracellular drug release via bilayer fluidity, which enables interaction with the peptidoglycan barrier and the establishment of a concentration gradient. Furthermore, niosomal vesicles enhance significant drug penetration into bacterial environments while protecting antibiotics from enzymatic degradation, thereby serving as an effective strategy to combat drug resistance [44]. Also, this encapsulation could result in improving the antimicrobial efficacy of the free drug via the prolonged release profile of niosomal drug delivery systems [20].

#### 2.2.2. Antibiofilm Study

**Figure 7:**
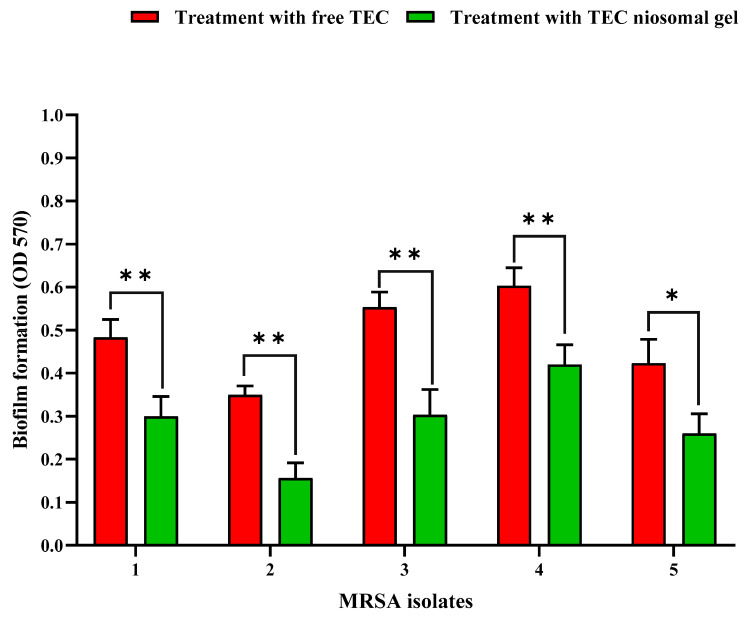
Comparison of anti-biofilm potential of free TEC and TEC niosomal gel against 5 MRSA strains (mean ± SD, *n* = 3, *: *p* < 0.05, **: *p* < 0.01).

In this research, the inhibitory efficacy of TEC niosomal gel against five MRSA biofilms was investigated and compared with a free TEC formulation. Our microtiter plate (MTP) results showed that treating the bacterial MRSA isolates with the TEC niosomal gel resulted in more suppression of biofilm production in comparison with the free drug (Figure 7). In fact, niosomes enable effective drug delivery to embedded bacteria by facilitating their diffusion into the biofilm matrix, thereby inhibiting biofilm formation. Also, the sustained release of drugs from niosomes enhances prolonged drug availability, which can help inhibit the development of resistance mechanisms in biofilm-forming bacteria [20].

### 2.3. Animal Study

#### 2.3.1. Wound Closure

**Figure 8:**
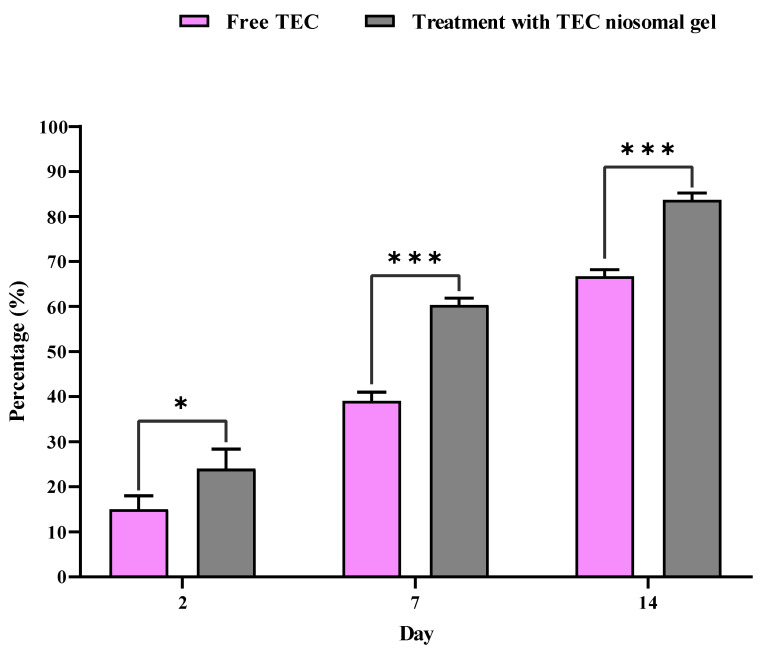
Comparison of closure percentage of wounds treated with free TEC and TEC niosomal gel during 21 days follow-up. * and *** represent a *p* < 0.05 and *p* < 0.001, respectively.

**Figure 9:**
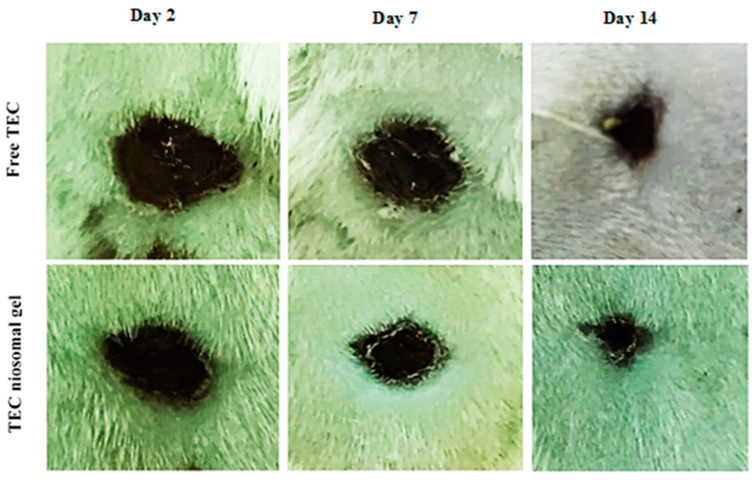
The macroscopic observations of infected wound created on the back skin of animal model on days 2, 7, 14.

The percentage of wound closure for non-healing infected wounds treated with the TEC niosomal gel was assessed on various post-dressing days: 2, 7, and 14 (Figure 8 and Figure 9). These data were compared to wounds that received treatment with free TEC. The group treated with the TEC niosomal gel demonstrated a significant enhancement in wound healing, with closure rates of 24.0%, 60.3%, and 83.6% on days 2, 7, and 14, respectively. In contrast, the free TEC group exhibited closure rates of only 15.0%, 39.0%, and 66.6% at the same time points (*p* < 0.05).

The limited penetration of topical medications into the skin layers poses a significant challenge for effective drug administration [45]. Additionally, several limitations, such as low absorption, water insolubility, and instability in vivo, result in inadequate delivery of the drug to the target organisms, contributing to the ineffectiveness of conventional dosage forms for treating wound infections [46]. Our study showed that the macroscopic observations of days 2, 7, and 14 demonstrated that treatment with the niosomal gel formulation had a significant effect on the re-epithelialization of full-thickness wounds. Indeed, antibiotic-carrying niosomes dispersed into HPMC and Poloxamer 407 polymers had a prominent antibacterial effect due to the efficient release of TEC at the infected site, which decreased the number of neutrophils. Conversely, the soluble components resulting from the degradation of the chemokine receptor (CXCR1) may activate toll-like receptor 2 (TLR2), leading to an increase in pro-inflammatory cytokines and the recruitment of excess neutrophils. Decreasing the number of neutrophils had a significant effect on the severity of the inflammatory phase. Therefore, niosomal gel could present as an effective delivery system for antimicrobial agents which has high potential to increase the local concentration of drugs at the site of injury.

#### 2.3.2. H&E Staining

**Figure 10:**
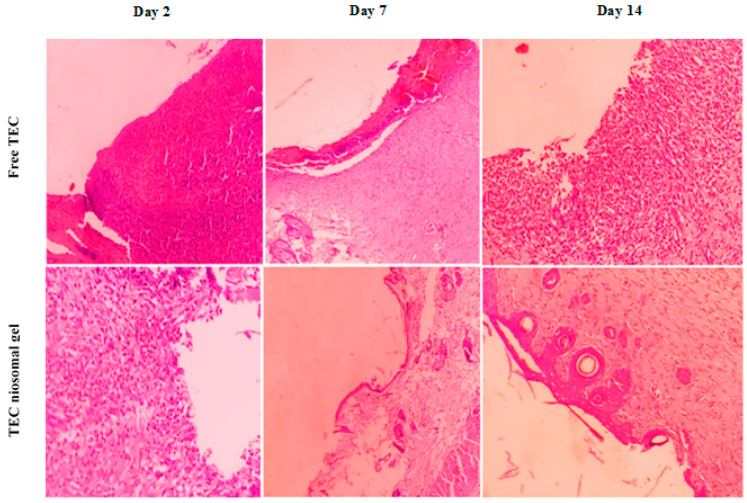
The histological evaluation of H&E stains of infected wounds in treated and un-treated groups on days 2, 7, 14.

A histological evaluation of hematoxylin and eosin (H&E)-stained slides was conducted, and the resulting images obtained through light microscopy are presented in Figure 10. Histological assessments of the infected wounds following treatment with the TEC niosomal gel were performed on days 2, 7, and 14 post-treatment, comparing the group treated with free TEC. Notable infiltration of inflammatory cells into the wound bed was evident in both groups on day 2. Re-epithelialization commenced at the wound margins; however, it was not fully achieved in either group. By day 14, the infected wounds treated with the TEC niosomal gel exhibited complete coverage by a new epithelial layer, whereas wounds in the free TEC group remained open. Also, the incomplete re-epithelialization and persistent inflammatory cells in the control group indicated that these wounds were still in the inflammatory phase and infected with MRSA isolates.

Niosome-based gel systems have the potential to enhance the transdermal delivery of drugs and improve the efficacy of antibiotics in the treatment of bacterial wound infections. In this context, Zaid Alkilani et al. demonstrated that niosomal gel enhanced the permeation characteristics of encapsulated drugs while offering controlled therapeutic activity and extended residence time. Additionally, the formulated niosomal gel exhibited superior antimicrobial effectiveness and physical stability against *S. aureus* compared to conventional gel formulations [19]. Additionally, Dharashivkar et al. indicated that the niosomal gel system could accelerate wound healing by enhancing the release profile of silver sulfadiazine (SSD). In this research, an SSD niosomal gel was evaluated for the topical delivery and treatment of burn wounds. The microbiological analysis in this study revealed that niosomal SSD exhibited greater anti-*S. aureus* activity compared to the commercially available dosage form. Furthermore, the animal study conducted by Dharashivkar et al. demonstrated that niosomal gel could improve the angiogenesis and re-epithelialization of damaged skin, positioning it as a promising alternative strategy for transdermal drug delivery [47]. Also, it was demonstrated that niosomal gels could develop into a favorable wound-healing agent in patients due to their spreadability property [48]. In addition, in the study by Kong and Colleagues, drug-embedded niosomal gel was presented as an antimicrobial system for local antibiotic delivery against burn infections [49]. Moreover, the bioadhesive properties of the prepared system are a crucial factor in wound dressing, as they facilitate prolonged retention and enhanced absorption of the drug at the application site. Consequently, incorporating the drug into a niosomal gel improves the pharmacokinetic profile of the encapsulated drug and presents a viable solution for topical therapy in bacterial wound infections, particularly those caused by *S. aureus* [42]. Dugal et al. demonstrated that the encapsulation of povidone-iodine (PVP-I) within niosomal carriers presents a suitable option for topical drug delivery by providing an improved sustained release profile for the drug. Furthermore, the anti-*S. aureus* efficacy of niosomal PVP-I offers a novel and promising approach for eliminating skin infections caused by antiseptic-resistant *S. aureus* isolates [50]. Furthermore, Mansouri Boroujeni et al. fabricated electrosprayed cefazolin-containing niosomes, where in vivo results confirmed the capability of the synthesized system to increase skin regeneration by tissue remodeling, improving re-epithelialization, and angiogenesis [51].

#### 2.3.3. Colony Count

**Figure 11:**
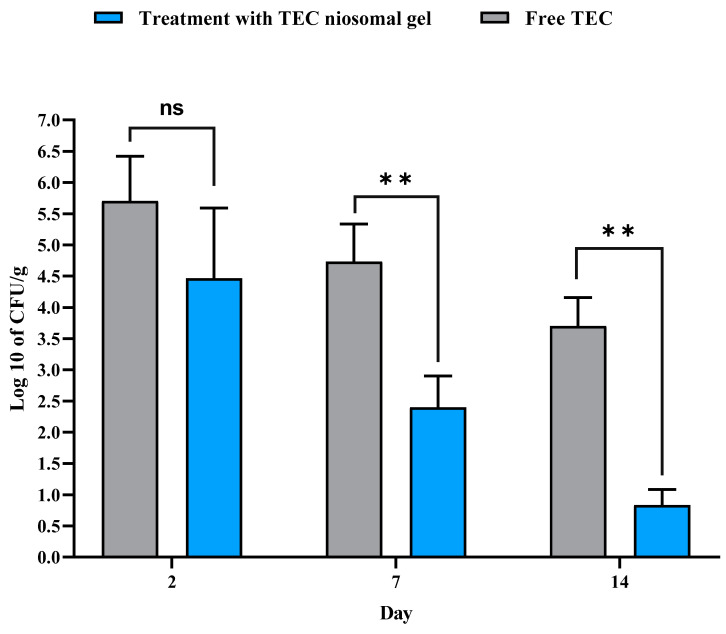
Comparison of the colony count (CFU/g) of wounds treated with free TEC and TEC niosomal gel in the tissue suspension collected from animals on days 2, 7, and 14 post-treatment (ns: not significant, ** represents *p* < 0.01).

The antibacterial efficacy of free TEC and the formulated niosomal gel on non-healing infected wounds is illustrated in Figure 11. Throughout all experimental time points (2, 7, and 14 days), a significant reduction in the number of bacterial colonies was noted in the wound beds treated with the TEC niosomal gel compared to those dressed with free TEC. On day 2, the colony count for wounds treated with the TEC niosomal gel was 4.46 × 10^8^ CFU/g, while the free TEC group exhibited a count of 5.7 × 10^8^ CFU/g (*p* > 0.05). On day 7, the bacterial counts were recorded at 2.4 × 10^8^ CFU/g for the TEC niosomal gel group and 4.7 × 10^8^ CFU/g for the free TEC group (*p* < 0.001). On day 14, bacterial counts were significantly lower in the TEC niosomal gel group at 0.83 × 10^8^ CFU/g compared to 3.7 × 10^8^ CFU/g in the free TEC group (*p* < 0.001). Furthermore, the application of the TEC niosomal gel on non-healing wounds infected with MRSA isolates resulted in a significant decrease in bacterial load within the wound bed when compared to wounds treated with the free formulation (*p* < 0.01).

As previously noted, post-wound infections caused by MRSA isolates represent a significant challenge in healthcare and dermatological settings. Prolonged hospitalization is a key risk factor for the development of MDR-infected wounds in clinical settings. Decreasing the number of the bacterial colony count is a key factor in alleviating the duration and severity of the inflamed skin. This reduction facilitates a more rapid transition to the proliferative and remodeling phases, thereby preventing the wound from becoming chronic [52]. Furthermore, the damaged area is filled more rapidly due to positive effects on fibroblasts, which promote increased collagen production. This process creates a moist environment that facilitates cell migration at the wound site [9,53]. In general, the progression from proliferation to the remodeling phase occurs with increased vascularization and granulation, resulting in complete wound healing in a rapid period of time. It can be hypothesized that HPMC nanofibrous structures have demonstrated effective results in promoting vascularization [54]. Therefore, the niosomal gel system can be a powerful transdermal nano-carrier and may provide a new perspective for treating bacterial skin infections. Indeed, niosomes demonstrate favorable interactions with human skin when utilized in topical formulations, particularly enhancing the characteristics of the stratum corneum. This improvement leads to a reduction in transdermal water loss and an increase in skin smoothness through the replenishment of skin lipids. While niosomes offer similar advantages, they are preferred over traditional lipid-based systems due to the higher costs and lower stability associated with lipids, which have been substituted with nonionic surfactants. Drug-loaded niosomes for dermal application interact with epidermal tissue without producing immediate or pronounced systemic effects. Consequently, wound dressings with the niosomal gel system exhibit excellent antibacterial activity, which can be a novel strategy for managing chronic wounds infected with MRSA strains. However, the possible potential of niosome-based gel systems to protect wounds from resistant pathogens needs further investigation.

## 3. Conclusions

This research indicated that niosomal gel significantly enhances the pharmaceutical properties of free TEC, suggesting its potential for delivering antimicrobial agents. Furthermore, the integration of the drug into niosomal gel augmented the antibacterial efficacy of the free drug, positioning it as a promising drug delivery system against clinical isolates of MRSA. Additionally, the in vivo results demonstrated that the niosomal gel delivery system had acceptable wound-healing activity, potentially mitigating MRSA challenges within healthcare settings. However, further studies are necessary to evaluate the antibacterial and anti-biofilm capabilities of the niosomes, which could provide valuable insights for the clinical advancement of this drug delivery system in combating bacterial infections.

## 4. Materials and Methods

### 4.1. Materials

Amicon Ultra centrifugal filter, methanol, Spectra/Por^®^ dialysis membrane (MWCO 12 kDa), and teicoplanin A2 (≥80%, HPLC) were provided by Sigma-Aldrich, Bengaluru, India. Span 60 (sorbitan monostearate), cholesterol, HPMC, Poloxamer 407, polyethylene glycol (mPEG 2000), all culture media, chloroform, and crystal violet were also purchased from Merck, Darmstadt, Germany. Additionally, the *S. aureus* ATCC 6538, as standard MRSA strain, was donated by the department of microbiology, Hamadan University of medical sciences, Hamadan, Iran.

### 4.2. Bacterial Isolation

In this study, a total of 5 MRSA biofilm-producing strains isolated from clinical specimens were used for in vitro and in vivo microbial analysis [28]. The biofilm-forming capability of MRSA strains was proved by MTP [18].

### 4.3. Preparation of TEC Niosomal Gel

In our study, the niosome vesicles were firstly synthesized using the thin film hydration method as outlined in the literature, with minor modifications applied [28,55]. Initially, a specific amount of Span 60, mPEG2000, and cholesterol, with a molar ratio of 2:0.06:1, was mixed with an organic solvent (chloroform–methanol solution in a ratio of 2:1 *v*/*v*) and magnetically stirred (50 min, 25 °C, 150 rpm). The solution was subsequently transferred to a round-bottom flask and gently spun at 150 rpm for 50 min at 60 °C, using rotary evaporation (WB Eco Laborota 4000 Model, Heidolph, Schwabach, Germany) to remove the organic phase. After forming a thin lipid film, the residual solvent was removed via purging with nitrogen for 10 min, and the dried lipid was resuspended in 20 mL of 1 M saline solution (PBS, pH 7.4) containing TEC for 45 min. For synthesizing niosomal gel, one part of the gel composed of HPMC (3%) and Poloxamer 407 (22%) was added drop-wise to four parts of the niosomal solution and mixed thoroughly on a magnetic stirrer for 60 min at room temperature [56,57]. Utilizing the same protocol, the blank niosomal gel was prepared without the incorporation of TEC. Following a visual assessment of the formulations for flocculation and turbidity, they were stored at 4 °C for future experiments. Table 1 represents the specific amounts and materials used in the TEC niosomal gel.

### 4.4. Evaluation of the Physicochemical Features of Niosomal Gel

#### 4.4.1. Morphological Evaluation

The morphology of synthesized niosomes and niosomal gel was examined using an FE-SEM (Hitachi S4160, Tokyo, Japan). Initially, 50 µL of the sample was mixed with 5 mL of distilled water for this analysis. Subsequently, 2 μL of the resulting suspension was placed onto a glass surface. After allowing the suspension to dry, a layer of gold was applied to the surface to mitigate electrostatic charging during the examination. Finally, the sample was analyzed using an image software [58,59].

#### 4.4.2. Hydrodynamic Size, Homogeneity, and Zeta (ζ) Potential

The hydrodynamic size, surface zeta (ζ) potential, and PDI of the niosomal formulation were evaluated by a zeta-sizer instrument (Horiba, SZ-100, Kyoto, Japan). To do this, a 1: 10 dilution of the samples was prepared and probe-sonicated (2 min, 25 °C, 30% Amp) before measurement of particle size and surface charge. Notably, the hydrodynamic size and zeta potential of niosomes were determined by dynamic and electrophoretic light scattering (DLS/ELS) methods, respectively [58].

#### 4.4.3. Drug Release Study

A dialysis method was employed using a spectrophotometer to quantify the concentrations of niosomal gel formulation, in accordance with established guidelines from the literature [60]. Initially, the un-entrapped TEC was separated by ultracentrifugation, and then the dialysis bag (cut-off range 12 kD) containing 1 mL of niosomal gel was placed in 25 mL of 5 mM PBS (pH~7.4) as recipient medium while being magnetically stirred for 48 h (150 rpm, at 37°), and 0.5 mL of recipient medium was collected at different intervals. The resulting supernatant was analyzed, and the drug concentrations were determined using a standard curve.

#### 4.4.4. EE%

The percentage of TEC encapsulated into niosomal gel was estimated after separation of un-entrapped drug by Amicon^®^ Ultra centrifugal filters (0.5 mL, cut-off: 50 kD). Briefly, 0.5 mL of niosomal gel formulation was placed in an Amicon tube and centrifuged for 20 min at 7500× *g* at 4 °C. The amount of TEC (un-entrapped TEC) in the liquid exiting the filter was calculated after measuring its optical density and estimating the TEC concentration using standard curve’s equation. Finally, the EE% of TEC in the niosomal gel was determined by the following formula [28]:

$$\mathrm{EE}\%=\frac{A-B}{A}\times 100$$

where “A” is the total amount of initially entrapped TEC, and “B” is the amount of un-entrapped TEC.

#### 4.4.5. Stability Analysis

The stability of the synthesized niosomes was evaluated by tracking changes in hydrodynamic size, EE%, and PDI over a period of 30 days at both 4 °C and 25 °C storage conditions.

### 4.5. Microbial Analysis

#### 4.5.1. MIC

The antibacterial effect of niosomal gel against MRSA isolates was performed using the microdilution protocol as recommended by CLSI [61]. Approximately 10^6^ CFU/mL of bacterial cells in the Mueller–Hinton broth (MHB) culture were exposed to different concentrations of the sample. After overnight incubation at 37 °C, the MIC was determined as the lowest concentration at which no visible growth of the bacterial inoculum was observed, as indicated by the absence of turbidity. The negative control consisted of an un-inoculated well, while the inoculated broth with MRSA 6538 served as a positive control.

#### 4.5.2. MBC

After a 24 h incubation period, 100 μL aliquots of each well that exhibited no visible bacterial growth were transferred onto the Mueller–Hinton agar (MHA) culture plates and incubated overnight at 37 °C. The plates were subsequently assessed for bacterial viability by observing microbial growth. The MBC values were determined when >99.0% of the bacterial inoculum was eliminated at the lowest concentration of the antimicrobial agent. Un-inoculated bacterial MHA medium and microbial strain (MRSA 6538) were used as negative and positive controls, respectively.

#### 4.5.3. Anti-Biofilm Activity

To analyze the anti-biofilm potential of niosomal gel, 200 μL of the bacterial tested suspension cultured onto tryptic soy broth (TSB) medium was seeded into a microtiter plate. Subsequently, increasing concentrations of sample were added to each well. Following overnight incubation of the microplate at 37 °C, the formed bacterial biofilms were washed and fixed via absolute methanol. At the end, the biofilms were stained with crystal violet (1.5% *w*/*v* solution), and the OD of each well was reported using a microplate reader (Titertek, R Multiscan, Pforzheim, Germany) at 570 nm [62,63,64].

### 4.6. In Vivo Study

#### 4.6.1. Creation of Full-Thickness Wounds Infected with MRSA

The in vivo antibacterial potential of the niosomal gel was assessed to investigate the healing of the animal wound model. All the animal procedures were performed based on the recommended protocols by the committee for laboratory animal care [65]. Additionally, the animal procedures received approval from the ethics committee at Hamadan University of Medical Sciences (IR.UMSHA.REC.1403.105), and were performed based on the ARRIVE guidelines. Male Wistar rats with 200 g weight and 6–8 weeks old were provided by the animal breeding center of Hamadan University of Medical Sciences and employed as the full-thickness excision model for wound-healing analysis. All animals were housed individually in ventilated cages under a controlled 12 h light and 12 h dark cycle, with unrestricted access to water and feeding. After acclimating, the animals were anesthetized through intraperitoneal injection with an appropriate dosage of xylazine/ketamine (Woerden, The Netherlands). Thereafter, the hair on the back skin was shaved and a full-thickness wound was formed using a sterile biopsy puncher. Subsequently, 100 µL of a bacterial suspension at a concentration of 3 × 10^8^ CFU/mL was injected subcutaneously into the exposed area. Then, the wounds were coated with a sterile bandage to maintain uniformity and prevent contamination. The administration of the synthesized niosomal gel and free agent was conducted every two days in an induced wound model using MIC dosing regimens. Notably, the infected wound model treated with free TEC was considered as the control group [66].

#### 4.6.2. Histological Observation

At 2-, 7-, and 14-days post-treatment, the animals were euthanized using high doses of xylazine and ketamine, which facilitated the easy scarification of rats after performing experiments. Skin tissue encompassing the wound bed, along with adjacent healthy skin, was harvested. All skin samples were fixed in formalin 10% for 72 h and subsequently dehydrated using a graded series of ethanol (30–100%). The samples were then embedded in paraffin blocks and sectioned into 5 μm thick slices for histological analysis. The slides were dyed with H&E to facilitate histological examination, and the wound-healing process was investigated by reporting pathological parameters [67].

#### 4.6.3. Macroscopic Observations

A photographic method was employed to assess the size of the wounds on days 2, 7, and 14 post-implantations. The percentage of wound closure was calculated using the following equation [1].

$$\mathrm{Wound}\;\mathrm{closure}\;(\%)=\frac{A-B}{A}\times 100$$

where “A” is the diameter of the wound on day 0, and “B” is the diameter of the wound on day 2, 7, or 14.

#### 4.6.4. Colony Count

At 2-, 7-, and 14-days post-implantation, the animals were euthanized as detailed subsequently. The skin sections were collected, weighed, and homogenized in 1 mL of sterile PBS (pH = 7.4). A volume of 100 µL of the homogenized tissues was inoculated into MHA medium and incubated overnight at 37 °C. Afterward, the number of bacterial colonies grown on the MHA plate was reported as CFU per gram (CFU/g) [67].

### 4.7. Statically Analysis

Analysis of variance (ANOVA) and the Tukey test were utilized to evaluate significant differences among the groups studied. The statistical analysis and graph creation were conducted using GraphPad Prism version 9.0. Notably, the differences in mean values reached a statistically significant level with a *p*-value of less than 0.05.

## Figures and Tables

**Table 1:** The composition of TEC niosomal gel formulation.

Material	Specific Amount	Molar Ratio
Span 60	251.8 mg	2
mPEG 2000	35.0 mg	0.06
Cholesterol	113.0 mg	1
Poloxamer 407	5.5 g	1.5
HPMC	750 mg	2
Teicoplanin	100 mg	0.20

## Data Availability

The data presented in this study are openly available in this article.
